# At Lunch with a Killer: The Effect of Weaver Ants on Host-Parasitoid Interactions on Mango

**DOI:** 10.1371/journal.pone.0170101

**Published:** 2017-02-01

**Authors:** Valentina Migani, Sunday Ekesi, Katharina Merkel, Thomas Hoffmeister

**Affiliations:** 1 University of Bremen, Faculty of Biology/Chemistry, Institute for Ecology, Bremen, Germany; 2 International Centre for Insect Physiology and Ecology(*icipe*), Nairobi, Kenya; 3 Queensland University of Technology, Brisbane, Australia; USDA-ARS Beltsville Agricultural Research Center, UNITED STATES

## Abstract

Predator-prey interactions can affect the behaviour of the species involved, with consequences for population distribution and competitive interactions. Under predation pressure, potential prey may adopt evasive strategies. These responses can be costly and could impact population growth. As some prey species may be more affected than others, predation pressure could also alter the dynamics among species within communities. In field cages and small observation cages, we studied the interactions between a generalist predator, the African weaver ant, *Oecophylla longinoda*, two species of fruit flies that are primary pests of mango fruits, *Ceratitis cosyra* and *Bactrocera dorsalis*, and their two exotic parasitoids, *Fopius arisanus* and *Diachasmimorpha longicaudata*. In all experiments, either a single individual (observation cage experiments) or groups of individuals (field cage experiments) of a single species were exposed to foraging in the presence or absence of weaver ants. Weaver ant presence reduced the number of eggs laid by 75 and 50 percent in *B*. *dorsalis* and *C*. *cosyra* respectively. Similarly, parasitoid reproductive success was negatively affected by ant presence, with success of parasitism reduced by around 50 percent for both *F*. *arisanus* and *D*. *longicaudata*. The negative effect of weaver ants on both flies and parasitoids was mainly due to indirect predation effects. Encounters with weaver ant workers increased the leaving tendency in flies and parasitoids, thus reduced the time spent foraging on mango fruits. Parasitoids were impacted more strongly than fruit flies. We discuss how weaver ant predation pressure may affect the population dynamics of the fruit flies, and, in turn, how the alteration of host dynamics could impact parasitoid foraging behaviour and success.

## Introduction

A major challenge in ecology is to understand the complex interactions that shape ecological communities, and to conceptualize the involved processes. Consumption (i.e. predation) and competition are considered to be two main forces structuring ecological communities [1–3]. Trophic relations are particularly interesting since communities can be represented as food chains of interacting resources, consumers, and predators. A growing literature focuses on predator-prey dynamics, disentangling important mechanisms underlying these interactions [4,5]. The main focus of this research has been on the effects of direct consumption by a predator on its prey. However, the impact of predation is not limited to lethal effects of predators upon their prey. In fact, the mere presence of a predator can induce changes in the expressed phenotype of the prey in order to avoid or reduce predation pressure. These changes include, amongst others, increased patch leaving probability [6], reduced foraging activity [7], enhanced movement to predator-free areas [8], and an increased tendency by the forager to search in low quality patches [9–11]. When compared to direct consumption itself, the costs associated with such changes can have an equal, or even higher impact upon the prey population growth [4, 12–14]. Moreover, they might affect not only the prey, but also the organisms directly linked to it, such as resources or competitors [15, 16].

According to optimal foraging theory, individuals should maximize their energetic intake, while minimizing the costs of foraging, such as predation risk [17]. The trade-off between benefits and costs of foraging forces foragers to optimize time allocation to resource patches based upon their skills to evaluate a given patch. As patch value can be influenced by predation risk, including predator presence in optimal foraging models is crucial to predict and understand decision making processes under predation pressure [18].

Despite predation being one of the most important shaping forces for ecological communities, there remains a lack of integrating optimal foraging decisions into studies on the influence of predation pressure in multi-species systems. Considering the effect of predation within assemblages of foragers/competitors may clarify if, and how, a forager´s decision under predation pressure influences the interactions among foragers, resources, and predators. Such multi-trophic interactions are particularly important for biological control programmes, which rely on foraging interactions between a pest and its natural predators. When local natural enemies are ineffective against a target pest, one of the most common biological control practices is the introduction of new predators or parasitoids in the areas of interest. However, the introduction of new species within a pre-existent community may alter the trophic interactions among the species involved. This could, in turn, lead to an increase in indirect trophic effects. When both generalist (e.g. mostly predators) and specialist (e.g. mostly parasitoids) natural enemies are used for biological control programmes, intraguild predation or apparent competition may occur, potentially impairing the success of the programme [19]. The past decades have seen an increasingly critical discussion regarding the use of both generalist and parasitoids to reduce pest species [20]. Predatory ants, for example, are generalist predators, ubiquitous within agricultural systems, providing important ecosystem services such as pest control [21]. As much as they can be beneficial against pests, with their polyphagy, they can also consume parasitoids, reducing the effects of these biocontrol agents on the target pest (reviewed in [22]). When parasitoids are released in new areas in order to establish natural populations in the field, the presence of such generalist predators may hamper the successful establishment of parasitoid populations. Clarifying whether the presence of a generalist predator may be harmful for the successful control by parasitoids can be crucial for an improved biological control planning and management.

Here, we studied the interactions between a generalist predator, the African weaver ant, *Oecophylla longinoda* (Latreille), two parasitoid species, *Fopius arisanus* (Sonan) and *Diachasmimorpha longicaudata* (Ashmead) and two fruit fly species that primarily infest mango fruits, *Ceratitis cosyra* (Walker) and *Bactrocera dorsalis* (Hendel). *Ceratitis cosyra* is a native sub-Saharan species, while *B*. *dorsalis*, first identified as *B*. *invadens*, is an invasive species in Africa. Due to its high polyphagy, *B*. *dorsalis* rapidly spread throughout most of the sub-Saharan African continent, affecting primarily mango fruits, with infestation levels reaching up to 80 percent. There is also evidence that *B*. *dorsalis* is outcompeting *C*. *cosyra* in the field [23, 24]. *Fopius arisanus* and *D*. *longicaudata*, two exotic parasitoids with Asian origins, were introduced in East and Central Africa in order to control and reduce fruit damage. *Fopius arisanus* is an egg-prepupal parasitoid species, while *D*. *longicaudata* parasitises the larvae of the fruit flies and hatches from the pupae. Additional to specialist parasitoids, the use of native generalist predators has also been explored. As natural inhabitants of mango trees, weaver ants have been found effective in reducing fruit fly infestation levels in mango orchards [25–27]. There is evidence that the ant cues deter the flies from ovipositing on the mango fruit [28]. However, as these ants are predators of insects in general [29], they may also interfere with the biological control effort of the introduced parasitoids. Recently, it has been shown that the mere presence of ant cues negatively affects oviposition success by the parasitoid *F*. *arisanus* [30].

We explored whether the ants interfere with fruit fly and parasitoid foraging behaviour, assessing fruit fly infestation levels and parasitism rate by the parasitoids in the presence of ants in semi-field conditions. We then investigated, through behavioural observations in a controlled environment (i.e. small Perspex cages), to what extent the interference with the weaver ants is due to indirect effects rather than direct consumption, as both the fruit flies and parasitoids can fly, and thus easily escape when attacked by the ants. Parasitoids and fruit flies use different strategies to forage, and thus may react differently to predation pressure. If this is the case, we expect the weaver ants to alter the relationship between fruit flies and parasitoids. Since foragers that display rapid host-handling behaviour are favoured in predator presence [10, 15], we expect the ants to have a different impact on fruit fly and parasitoid species, respectively. In particular, as *B*. *dorsalis* and *C*. *cosyra* differ in probing duration, we expect the weaver ants to have less of an impact on the forager with the shorter probing time. As the parasitoid species differ in the host stage they parasitise, they might also differ in the magnitude of the impact of the weaver ants.

Specifically, we tested (1) if weaver ants interfere with fly or parasitoid foraging behaviour on fruits, (2) if and to what degree the ants impact oviposition behaviour in both the flies and their parasitoids, and whether this impact differs (3) between flies and parasitoids, (4) between the two fly species that differ with respect to their time budgets when ovipositing, and (5) between the two parasitoid species. We discuss how weaver ants may interfere and alter the trophic dynamics among and between fruit flies and parasitoids.

## Materials and Methods

We carried out two sets of experiments at the International Centre of Insect Physiology and Ecology (*icipe*), Nairobi, Kenya, hence forth named as “field cage-” and “observation cage experiment”. The field cage experiment aimed at elucidating if the presence of weaver ants would impact mango infestation caused by the fruit flies and the foraging efficiency of the parasitoids on infested mangoes under semi-field conditions. Since we could not study the nature of the interaction, i.e., direct with lethal encounters or indirect across the time period that cohorts of a single species of flies or parasitoids were exposed, we used experiments in small observation cages to observe behavioural changes of individual fruit flies or parasitoids in the presence and absence of ant workers. All experiments were carried out under ambient light and temperature conditions. Replicates for different treatments were interspersed and arranged such that time and day effects could not contribute systematically to the differences between treatments.

### Fly and parasitoid rearing

Puparia of *B*. *dorsalis* and *C*. *cosyra* were obtained from a main colony reared at the Animal Rearing and Containment Unit (ARCU) of *icipe*. The stock cultures originated from natural populations collected from infested mango fruits purchased at a local market in Nairobi, Kenya. Flies were reared according to the methodology described by Ekesi and colleagues [31] and kept at lab conditions of 28±1°C and at a relative humidity (RH) of 50± 8%, with a photoperiod of L12:D12. The colony had already been maintained for more than 100 generations at the time of our experiment. Every 6–12 months the colonies were rejuvenated by the introduction of wild flies.

*Fopius arisanus* and *D*. *longicaudata* were obtained from a main colony reared at *icipe*. The initial cohorts were obtained from the University of Hawaii in Manoa, Honolulu, Hawaii, in 2006 and at the time of the experiments had been maintained for 99 generations for *D*. *longicaudata* and 97 for *F*. *arisanus*. Both species were reared according to the methodology reported by Mohamed and colleagues [32] and kept at lab conditions of 25±1°C, with a photoperiod of L12:D12.

### Ant collection and colony establishment

Colonies of the weaver ant, *O*. *longinoda*, were collected at the *icipe* field station in Muhaka, Coast Province, Kenya, by cutting the branches with nests from trees. No special permission was required for nest collection, as the African weaver ant is not an endangered species and we did not collect from private property. A single polygynous weaver ant colony can count several nests on the same or neighbouring trees. In order to reduce the effect on individual colonies with our collection, we took only one or two nests per tree. Subsequently, the nests were carried to the *icipe* headquarter in Nairobi. For transportation, the nests were stored in plastic containers (45x30x15cm) covered with fine netting material and closed with a ventilated lid. Once in Nairobi, the nests were placed on branches of *Ficus benjamina* L. (Moraceae) and *Schefflera* spp. Forst (Araliaceae) maintained in greenhouses at ambient temperature. All colonies were provided with a saturated sugar-water solution every day. Thrice per week all colonies were supplied with protein food, consisting of 10–12 larvae of fruit flies (2^nd^ instar), or small pieces of raw fish (*Tilapia* spp.) placed in Petri dishes on the branches of the plant. When colonies were used for field cage experiments, they were provided with the sugar-water solution only. We managed to establish 20–25 weaver ant colonies. Colonies were rejuvenated through new collections every 6–8 months.

### Field cage experiment

Field cage experiments were set up to study if the presence of weaver ants had any impact on the level of mango infestation caused by the fruit flies, and on the rate of parasitism achieved by the exotic parasitoid species under semi-field conditions. For limitations in space and handling capacity, we repeatedly used four screen houses (h x w x l: 2x1.50x1.50m), placed in a two by two square grid. Two screen houses contained two plants (*Schefflera* spp.), each with established colonies of the African weaver ants, while the other two, used as control, contained two uncolonised plants (*Schefflera* spp.). Which of the cages would receive a treatment or control was determined by a random draw. When one of the cages needed repairing, only two trials, one treatment and one control, were run simultaneously. Treatment and control were exchanged between screen houses every 7–10 days by moving plants with ants to field cages that had housed control plants and vice versa, so that all the screen houses hosted trials with ants and without ants (i.e. control). To control for any possible effect of individual screen houses, screen house identity was used as random term in statistical models. A total of 100 replicates was performed: 26 replicates were run per each of the two fruit fly species and 24 replicates per each of the two parasitoid species. For each trial two mango domes (i.e. half cut mango fruit with the seed and pulp carefully scooped out) per screen house were randomly placed on the branches of the plants as oviposition substrate. Trials were run for two consecutive days. One day was left between trials to clean the screen houses from the previously used insects (i.e. flies or parasitoids). For both fly and parasitoid trials, fifty females of a single species of either fruit flies or parasitoids were released on the first day. In order to maintain a constant number of insects in the screen cages and replace the ones that could have died or escaped, twenty females of the same species were released on the second day. The species tested were rotated each week in order to avoid having a single species tested within a single time period. For *B*. *dorsalis* and *C*. *cosyra*, each trial lasted for around 10 hours (from 8 am to 6–6.30 pm) after release. Then, the mango domes were collected and the number of eggs laid by the flies recorded. For *F*. *arisanus*, each mango dome was previously infested with 50 eggs of *B*. *dorsalis*. In order to avoid egg desiccation, another mango dome was mounted underneath the infested one. Each trial with parasitoids lasted for 24 hours. After the trial, the domes were collected, washed and the fly eggs transferred on black cotton clothes placed in Petri dishes. First-instar larvae were collected from the Petri dishes and mounted on microscope slides with De Faure’s fluid, which digests the larval body fat and allows the parasitoid egg to be easily seen under the microscope, if present. The eggs that were not hatching after the first day of incubation were dissected. For *D*. *longicaudata*, each dome was previously infested with 20 second-instar larvae of *B*. *dorsalis*. Again, each trial lasted 24 hours. Then, the mango domes were collected and the larvae dissected to examine for parasitism.

#### Data analysis of field cage experiments

We used R version 3.2.1 [33] for the statistical analysis and for creating the graphs. To analyse the effect of the presence of weaver ants on fly oviposition behaviour, the number of eggs laid by the flies as a function of the presence or absence of ants in the cage was tested using Generalized Linear Mixed Models (GLMMs), included in “lme4” package [34], with Poisson distributed error. We grouped the trials run across two consecutive days together to correct for using the same insects for two consecutive days. Thus, trials and cage identity were used as random term to correct for repeated measurements. Data observations were used as additional random factors in the model to correct for overdispersion in the data [35]. For the parasitoid species, we tested the effect of ant presence on parasitism rate, i.e. the proportion of fly eggs for *F*. *arisanus* and fly larvae for *D*. *longicaudata* that were successfully parasitized with the total number of fly eggs/larvae present in the dome representing the binomial totals. These data were analysed using GLMM with binomial distributed error, corrected for overdispersion in the data. To analyse if *C*. *cosyra* and *B*. *dorsalis* were differently affected by the presence of weaver ants in the field cages, we considered the number of eggs laid by flies of a given species as a function of the species and the presence and absence of weaver ants, and tested it using GLMM with Poisson distributed error. To test as well if the weaver ants had a different effect on the parasitism rate of *D*. *longicaudata* and *F*. *arisanus*, we compared the proportion of parasitized hosts as a function of parasitoid species, and the presence and absence of ants in the screen cages, and tested it using GLMM with binomial distributed error, corrected for overdispersion.

### Observation cage experiment

In order to clarify if the weaver ants could interfere with fruit fly and parasitoid foraging behaviour due to direct predation or through indirect effects, we set up a cage experiment which allowed us to carry out direct behavioural observations. A plant with an established colony of *O*. *longinoda* was connected through a metal wire to a 12x12x12 cm Perspex cage, which was placed on a wooden stool in order to be on the same level as the plant foliage. The wire was connected to a branch used by patrolling workers. In order to prevent other predators to crawl on the plants and attack the colonies, both the plant and the wooden stool were placed on trays of soapy water. Additionally, the cage on the wooden stool was suspended on a basin of soapy water. Ant workers were attracted to visit the cage by placing food baits (5–6 fruit fly 2^nd^ instar larvae) in each cage every morning for an acclimatization period of 3–5 days, in which no experiment was run. After that, the cage was left without baits for a day and the ant activity (visit to the cage) was monitored. Ants were provided with baits again before the experiments to increase visiting activity.

Mango domes were used for both fruit fly and parasitoid behavioural observations. However, for the parasitoids the mango domes were infested prior to the experiments with the respective hosts as follows: for *F*. *arisanus*, each mango dome was prepared with 3 artificial punctures distributed on the skin and the fruit was then exposed in a cage with *B*. *dorsalis* flies for 10 minutes. The mango was then collected and cleaned of excess eggs, so that only 10 eggs remained in each of the three punctures. For *D*. *longicaudata*, mango domes were prepared introducing ten 2^nd^ instar larvae either of *B*. *dorsalis* or *C*. *cosyra* in a half of the mango, from which the pulp was previously removed. To prevent the larvae escaping from the dome, a second mango dome was placed under the first and their edges were sealed by moulding clay.

Each mango dome was placed in a Petri dish, filled either with sand or cotton wool in order to prevent (i) the fruit flies from ovipositing at the edge, and (ii) the parasitoids from going to the bottom of the fruit. The dome was then placed in the centre of the cage with a metal wire connecting the cage to the plant holding the ant colony, allowing ant workers to enter the cage. A few minutes after the mango placement (this time was necessary for the ants to start again their normal activity inside and outside of the cage), a female fruit fly (18- to 21-day-old for *B*. *dorsalis* and 14- to 18-day-old for *C*. *cosyra*), or a female parasitoid (9- to 14-day-old *F*. *arisanus* and 13-to17-day-old for *D*. *longicaudata*), previously experienced with oviposition, was released into the cage and the recording of the behavioural observation started. The following events were recorded using The Observer software [36]:

- the duration and frequency that female flies/parasitoids visited the mango dome. We used this to calculate the time that females spent on the mango dome;
- the duration and frequency that flies spent probing the fruit with their ovipositor. This was not considered for the parasitoids;
- the time females spent ovipositing, i.e. laying their eggs into the fruit for the fruit flies and into the hosts for the parasitoids;
- the number of workers present in the cage;
- the number of weaver ants present on the mango;
- encounters in the cage, i.e. all the episodes in which the female and the ants met in the Perspex cage, but not on the mango dome, resulting in the female taking off. The ant usually displayed an “aggressive posture” (*sensu* [37]), came in contact with, touched and/or attacked the female in the cage; however, there were few episodes in which the fruit fly/parasitoid female took off without the ant worker displaying such posture;
- encounters on the mango, as above, but restricted to events occurring on the mango dome.

When an encounter was deadly for the foraging female, the observation was carried on for half an hour, continuously recording the number of workers in the cage and on the mango, to measure ant activity. At the end of each observation, the female fruit fly or parasitoid and the mango dome were carefully removed from the cage. If oviposition had occurred, the mango dome was dissected. For the fruit flies, the number of eggs laid in the dome were counted, while the hosts (either fruit fly eggs or larvae) were dissected to count the number of eggs laid by the parasitoids. One observation per colony per day was carried out. After 10–15 days, the colonies used for the experiments were replaced with new ones to avoid acclimatisation effects. All the observations started at 11am-12 pm, when the colonies reached the peak of their activity, and carried on not further than 5 pm, before the activity started decreasing [38, 39].

The same cage visited by the ants was used to run control trials. In this case, the metal wire was removed to prevent the ants entering the cage. The cage was placed on a table in the greenhouse, in order to maintain the same condition of the treatment, but to avoid the workers climbing the cage walls. Except for the absence of ants in the cage, all the other procedures of the above-described experiment were applied.

Observations were repeated for at least 20 replicates for control and non-control (i.e. with ants) experiments per each parasitoid or fruit fly species.

#### Data analysis of observation cage experiments

To test if the number of ants on the cage surface as well as on the mango dome has an effect on the time that observed fruit flies and parasitoids, respectively, spent on the fruit, we calculated the number of ants present in the cage and on the mango per second. We multiplied the number of ants in the cage or on the mango for the time interval (in seconds) in which this number did not change. Thereby, we obtained the number of ant workers per second, summing up the values and dividing the results by the total time of the observation. We used the same approach explained above to obtain the number of encounters in the cage and on the mango per second.

To assess the effect of the number of ants and the encounters on the proportion of the observation time that females spent on the fruit, we tested all these variables in a GLM with binomial error distribution. This was analysed for *B*. *dorsalis*, *C*. *cosyra*, as well as for *F*. *arisanus* and *D*. *longicaudata*. We considered only the observations in which the ants were visiting the cages. We fitted a model with all factors and 2-way interactions. We applied a stepwise backward deletion procedure to obtain the minimum adequate model, removing the least significant term.

To estimate how behavioural events occurring on mango domes affect the residence time of a forager on the dome in the presence of foraging ants, we used a Cox proportional hazards model, included in the package “survival” [40]. This model analyses how the included factors influence the tendency to leave the patch [41, 42]. We organized the data starting from the time a female landed on the dome until she left the fruit. We included ovipositions, encounters with ants, and the number of ants present on the mango dome at any moment in time as time-dependent covariates. Given that a female was visiting the fruit more than once (i.e. fruit re-visit), we reset the previous variables, starting from zero again. We considered the number of encounters that had already taken place with ants on the inner cage surface and fruit re-visits as time-independent covariates. We then tested the effect of the above-mentioned covariates on fruit leaving probability by implementing a Cox regression model. We included and tested the interaction between the encounters with ants in the cage and on the mango, as well as between the encounters with ants in the cage and the re-visit on the fruit. We optimised the model by removing the non-significant terms, in order to find the best model explaining our data.

An analysis across all the species was run to test for differences of the effect of the weaver ants using a GLM with binomial distributed error, corrected for overdispersion. We tested the proportion of time spent on the mango and the proportion of time spent for oviposition when ants were present across all the species.

To analyse if the two fruit fly species were differently affected by the presence of the ants, we tested the proportion of time that females spent visiting the fruit, probing, and ovipositing, in the presence and absence of weaver ants using a GLM with binomial distributed error, corrected for overdispersion. To test if the impact of weaver ants differed in the parasitoid species we used GLMs with binomial distributed error, and the proportion of time spent visiting the fruit or ovipositing as dependent variables.

## Results

### Field cage experiment

**Fig 1:**
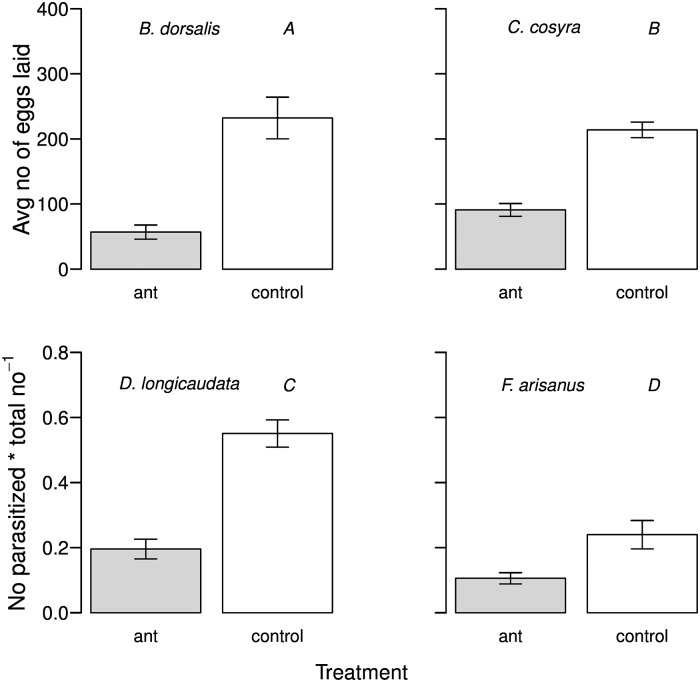
Fruit fly oviposition and rate of parasitism as a function of the presence of weaver ants. Comparison between the number of eggs for the fruit fly species (A and B) and the proportion of parasitized eggs/larvae for the parasitoid species (C and D) in the presence (grey bars) and absence (white bars) of ants in field cage experiments. The bars represent the average number of eggs laid for the fruit flies and the proportion between the number of parasitized eggs/larvae and the total number of fruit fly eggs/larvae present in the fruit.

For both *C*. *cosyra* and *B*. *dorsalis*, the presence of ants reduced the number of eggs that the females laid in the mango, with mangos in control tests (i.e. weaver ant absent) holding 4 times more eggs for *B*. *dorsalis* and 2 times more eggs for *C*. *cosyra* (*B*. *dorsalis*: χ^2^ = 10.74, d.f. = 1, n = 26, P = 0.001; *C*. *cosyra*: χ^2^ = 38.44, d.f. = 1, n = 26, P< 0.001; Fig 1A and 1B). Weaver ants also negatively affected the parasitism rate in both parasitoid species. The parasitism rate in *D*. *longicaudata* was reduced to about 17 percent in ant presence from 54 percent when ants were absent, while for *F*. *arisanus* it decreased from about 20 percent to about 9 percent with weaver ants present (*D*. *longicaudata*: χ^2^ = 36.18, d.f. = 1, n = 24, P< 0.001; *F*. *arisanus*: χ^2^ = 12.08, d.f. = 1, n = 24, P = 0.0003; Fig 1C and 1D). As single species were assessed separately, we could perform a two-by-two comparison among the two fruit fly species and the parasitoids, respectively, to check for differences in response to the presence of weaver ants. When comparing the effect of the weaver ants on the egg laying performances of the fruit flies and the parasitoids, we found no difference for *C*. *cosyra* and *B*. *dorsalis* (treatment x species: χ^2^ = 2.15, d.f. = 1, P = 0.14), but found that *D*. *longicaudata* was less affected by ants in its parasitism success in comparison to *F*. *arisanus* (treatment x species: χ^2^ = 19.07, d.f. = 1, P< 0.001).

### Observation cage experiment

**Fig 2:**
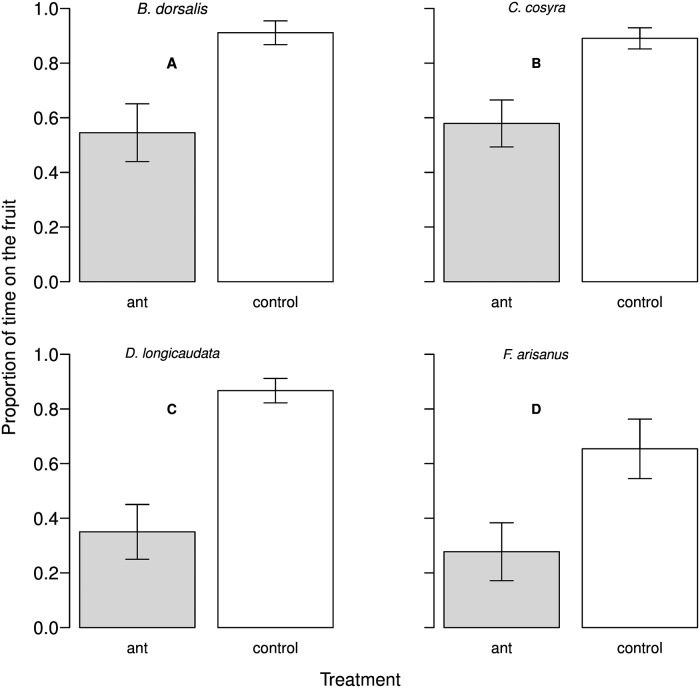
Time allocation to fruits by fruit flies and their parasitoids as a function of the presence of weaver ants. Proportion of time that both female flies (A and B) and parasitoids (C and D) spent on the fruit in the presence (grey bars) and the absence (white bars) of weaver ants during the observation cage experiments. The bars represent the proportion with the 95 percent confidence interval.

The proportion of time that females spent on the fruit was reduced when ants were present for both fly and parasitoid species (*B*. *dorsalis*: F_1,45_ = 36.10, P< 0.001; *C*. *cosyra*: F_1,54_ = 43.68, P< 0.001; *D*. *longicaudata*: F_1,58_ = 68.93, P< 0.001; *F*. *arisanus*: F_1,58_ = 21.06, P< 0.001; Fig 2). In line with this, ant presence negatively affected the proportion of time that females spent ovipositing (*B*. *dorsalis*: F_1,45_ = 22.02, P< 0.001; *C*. *cosyra*: F_1,54_ = 33.27, P< 0.001; *D*. *longicaudata*: F_1,58_ = 37.11, P< 0.001; *F*. *arisanus*: F_1,58_ = 4.65, P< 0.05).

**Fig 3:**
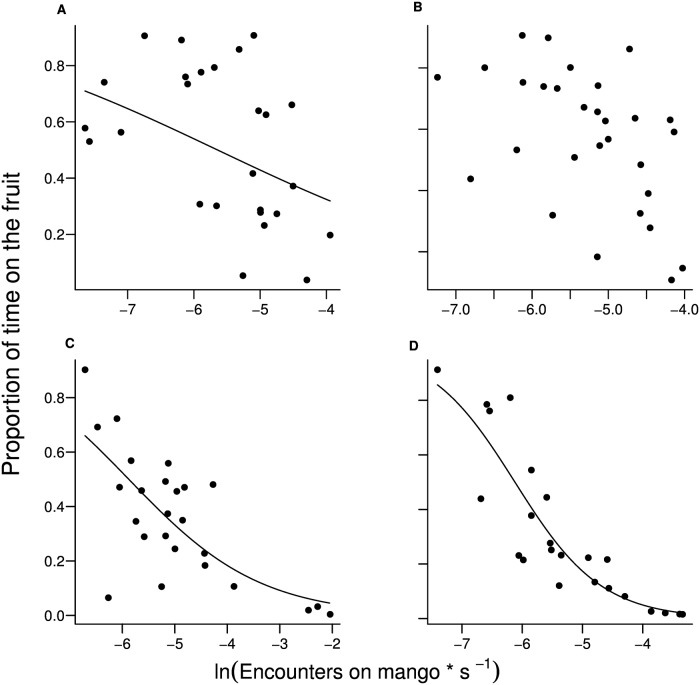
Time allocated to a mango fruit by fruit flies and their parasitoids as a function of encounters with weaver ant workers. Proportion of time that females spent on the mango dome as a function of the encounters with weaver ants on the fruit in *Bactrocera dorsalis* (A), *Ceratitis cosyra* (B), *Diachasmimorpha longicaudata* (C) and *Fopius arisanus* (D). The points represent the number of encounters per second within a single observation, transformed as a natural logarithm, and the line of the minimum adequate model as calculated by GLM with binomial distributed error, corrected for overdispersion (quasi binomial).

We observed the weaver ants actively catching and killing an individual fruit fly or parasitoid only once for *B*. *dorsalis* (26 observations in presence of ants), none for *C*. *cosyra* (32 observations), twice for *F*. *arisanus* (30 observations) and 3 times for *D*. *longicaudata* (32 observations). Thus, the effect of direct predation by the ants on both fruit flies and parasitoids was small. Encounters between the female and the ants on the fruit decreased visit duration for *B*. *dorsalis* and both parasitoid species (*B*. *dorsalis*: F_1,24_ = 5.22, P< 0.05; *D*. *longicaudata*: F_1,23_ = 24.8, P< 0.001; *F*. *arisanus*: F_1,21_ = 81.4, P< 0.01; Fig 3), however, there was no effect of ant encounters on the time that *C*. *cosyra* females spent on the fruit (F_1,29_ = 3.63, P = 0.67). When no encounters with ants occurred, the females spent approximately 66 percent of their time on the fruit in *B*. *dorsalis*, 45 percent in *D*. *longicaudata*, and 39 percent in *F*. *arisanus*. With only one encounter with the ant workers, the time spent on the fruit was reduced to 33.4 percent in *B*. *dorsalis*, to 34 percent in *D*. *longicaudata*, and to 17.6 percent in *F*. *arisanus*.

**Fig 4:**
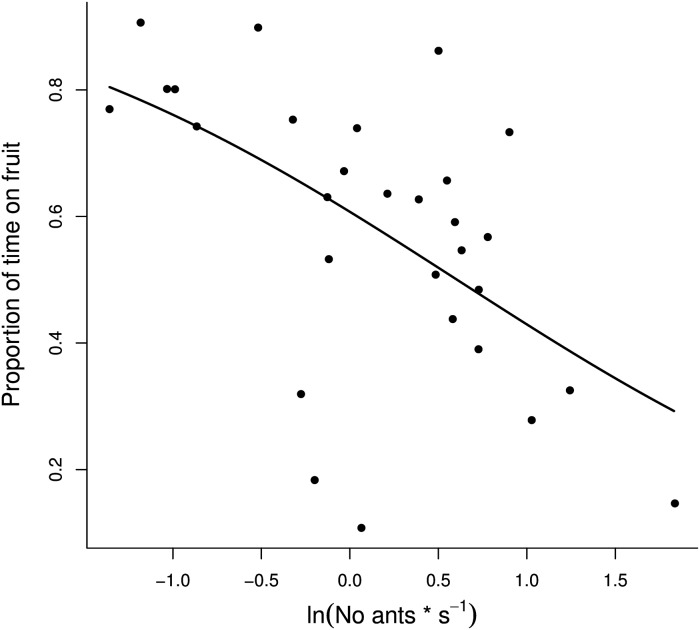
Time allocated to a mango fruit by *Ceratitis cosyra* as a function of the number of weaver ants present. Effect of the number of ants present on the mango per second on the proportion of fruit visit duration for *Ceratitis cosyra* as calculated by GLM with binomial distributed error, corrected for overdispersion (solid line). Data points represent the number of ants on the dome per second for each fly observation, transformed as a natural logarithm.

In *C*. *cosyra* the number of ant workers present on the mango had a negative effect for the proportion of the time that females stayed on the mango dome (*C*. *cosyra*: F_1,27_ = 12.16, P< 0.01; Fig 4), but this was not significant for the other species (*B*. *dorsalis*: F_1,24_ = 0.87, P = 0.36; *D*. *longicaudata*: F_1,23_ = 0.12, P = 0.73; *F*. *arisanus*: F_1,21_ = 0.65, P = 0.43).

In both parasitoid and fruit fly species the ant encounters on the mango increased the probability of fruit leaving (Table 1). However, while no other factor affected the tendency that a female leaves the mango for parasitoids, for the fruit flies, *B*. *dorsalis* and *C*. *cosyra*, probing activity decreased the probability of fruit leaving (Table 1). In *C*. *cosyra* the number of consecutive visits to the same fruit did not have any effect on the leaving tendency, but in *B*. *dorsalis* each previous fruit visit increased the likelihood that a female leaves the patch on a re-visit. In *B*. *dorsalis* neither the number of encounters between the individual and the ants in the cage nor the number of ant workers present on the mango dome had any effect (Table 1). In contrast, for *C*. *cosyra* the encounters between the ants and the flies in the cage (i.e. not on the fruit) decreased the probability of fruit leaving, while the number of ant workers present on the mango dome increased the tendency to leave the fruit. The latter result supports the findings obtained in the GLM analysis. Furthermore, we found a significant two-way interaction between the ant encounters in the cage and on the fruit decreasing the probability that a female *C*. *cosyra* leaves the mango.

**Table 1:** Factors affecting the latency to leave the fruit in presence of weaver ants.

Species	Factors	β coefficient	Relative risk ^a^ (95% confidence interval)	P ^b^
***Bactrocera dorsalis***	Probing	-0.052	0.94 (0.91–0.99)	< 0.01
	Encounters on mango	1.32	3.74 (2.39–5.83)	< 0.001
	Fruit re-visit	0.12	1.13 (0.98–1.30)	< 0.01
	Encounters in the cage	0.03	1.03 (0.91–1.15)	0.66
	No. of ants on mango	0.05	1.05 (0.91–1.21)	0.49
***Ceratitis cosyra***	Encounters in the cage	-0.096	0.91 (0.73–1.13)	< 0.05
	Encounters on mango	0.87	2.38 (1.56–3.64)	< 0.001
	No. of ants on mango	0.20	1.22 (1.07–1.39)	< 0.01
	Probing	-0.39	0.67 (0.52–0.89)	< 0.01
	Encounters cage x encounters on mango	-0.21	0.81 (0.67–0.98)	< 0.05
***Diachasmimorpha longicaudata***	Encounters on mango	1.22	3.40 (2.62–4.40)	< 0.001
***Fopius arisanus***	Encounters on mango	2.99	19.92 (10.71–37.06)	< 0.001

Parameter estimates and beta-values for the effects of probing, ovipositing, encounters on mango and in the cage, and fruit revisit on the likelihood of fruit leaving for fruit flies and parasitoids, when ants are present. Only the factors tested in the minimum adequate model are presented.

^a^ exponential (β)

^b^ P value from Anova (Type II) test.

We looked for differences between all the species we studied in response to the presence of the weaver ants, finding that both parasitoid females suffered a stronger reduction in the time allotted to visiting the fruit compared to fruit fly females when ants were present (species x treatment: F_3,213_ = 2.98, P< 0.01). In particular, *F*. *arisanus* females spent proportionally less time on the fruit compared to both *B*. *dorsalis* and *C*. *cosyra*, while *D*. *longicaudata* females had shorter mango visits in comparison to *C*. *cosyra*. When weaver ants were present, *C*. *cosyra* females spent about 58 percent of the total observation time on the fruit, *B*. *dorsalis* about 49.3 percent, whilst for *D*. *longicaudata* and *F*. *arisanus* it was only about 36.5 percent and 27.5 percent, respectively. To elucidate whether the oviposition behaviour across species was differently affected by weaver ant presence, we tested if the number and/or duration of oviposition events varied. We did not find any difference between the species (number of oviposition events: F_3,213_ = 1.08; P = 0.35; oviposition duration: F_3,213_ = 1.94; P = 0.13). This result was probably due to the low number of oviposition events that we could record in our cage observations. Nevertheless, we found a difference between the species when looking at the number of times that a female re-visited the fruit (F_3,213_ = 3.35; P< 0.05), with *F*. *arisanus* showing a lower tendency to re-visit than *B*. *dorsalis* and *C*. *cosyra* females.

## Discussion

### Effect of weaver ants on fruit fly infestation and parasitism rate

The aim of this work was to elucidate to what extent a generalist predator such as the weaver ant may interfere with foraging activities of both, the mango fruit flies and their exotic parasitoids, thereby possibly impairing the impact of the parasitoids on the flies. Previous studies suggest that weaver ants can severely impact fruit fly populations in the field [25–27]. Our field cage experiments confirmed these results [28], showing that weaver ants reduced the number of eggs laid by both *B*. *dorsalis* and *C*. *cosyra* (Fig 1A and 1B). Moreover, we showed that weaver ants can also decrease the success in parasitising hosts for both *D*. *longicaudata* and *F*. *arisanus* (Fig 1C and 1D), which is in line with previous findings [30]. In particular, egg laying was reduced by 50 percent for *C*. *cosyra* and by 75 percent for *B*. *dorsalis*, while percentage parasitism was reduced by 50 percent for *F*. *arisanus* and by 36 percent for *D*. *longicaudata*. Weaver ants (genus *Oecophylla*) have been identified as successful biological control agents for several pests that attack cocoa, mango, and cashew plantations both in Africa, in Asia, and Australia [43]. However, our experiments showed that *O*. *longinoda* workers negatively impact both exotic parasitoid species used for fruit fly biocontrol. In general, predatory ants can consume parasitoids or other predators that attack a target pest, disrupting the effectiveness of biological control strategies [8]. As *D*. *longicaudata* and *F*. *arisanus* are released in the areas of interest in order to establish populations of these parasitoids in the field, the presence of weaver ants may be detrimental to their successful establishment.

### Fruit fly and parasitoid foraging strategies in ant presence

Another important question addressed here was whether the weaver ants act as direct or indirect predators, i.e. whether they are actively predating and killing female fruit flies and parasitoids, or if they chase away the adult insects from the fruits through their aggressive behaviour. Direct consumption usually has an immediate effect on prey population density changes, while indirect predation can have delayed consequences that may be as detrimental, or even more, than those caused by direct consumption [12, 14]. Whilst direct consumption may affect only the individuals that are killed, and thus the number of organisms of the population that are encountered and killed in total is limited, indirect predation may affect a larger number of individuals and cause longer-lasting effects [13]. Suraci and colleagues [44] show how the fear of predation affects the population of its direct prey, and of the organisms the prey is feeding on, in a carnivore-mesocarnivore system. In particular, the broadcasting playbacks of wolf and puma (carnivores) causes a reduction of the racoon’s (mesocarnivore) foraging activity, and thus have a positive cascading effect on the population of the racoon’s prey. Differentiating between direct consumption and indirect predation effects of the weaver ants on fruit flies and parasitoids may be crucial for an improved ability to predict population dynamics and the potential spatial distribution of both the pest and natural enemy populations.

Only in less than 1 percent of the total direct observation experiments that were carried out, were the weaver ants observed actively catching and killing either the foraging fruit fly or the parasitoid female. Thus, the impact of direct predation by the weaver ants can be considered minimal. In contrast, our findings showed that weaver ants had an indirect predation effect on both fruit flies and parasitoids: encounters with patrolling ants increased the leaving tendency in fruit flies and parasitoids, thus reducing the time that the foragers spent on a mango. According to optimal foraging models, a forager should balance the trade-off between benefits (i.e. successful foraging) and costs (i.e. predation risk) associated with foraging activities [45]. Several studies present how a forager must make decisions in order to allocate the time spent foraging [41, 46–48]. There is evidence that indirect predation and predator pressure increase the costs of foraging and consequently decrease the quality of a patch [6,11]. Roitberg and colleagues [6] show both theoretically and experimentally that at high predation risk on the patch and moderate danger while travelling the tendency of patch leaving for a foraging parasitoid increases with decreasing patch value, making predation pressure a limiting factor for foraging activity. As for both parasitoids and fruit flies patch residence time is directly linked to foraging success, and in turn to reproduction [49, 50], a reduction in fruit visit duration by the flies and the parasitoids should negatively impact the chances to successfully oviposit, thus decreasing offspring production. In our experiments weaver ants reduced the time females spent on the fruits to 25 percent for *D*. *longicaudata*, and to 50 percent for both *B*. *dorsalis* and *F*. *arisanus*, which should translate into a negative influence on the number of offspring produced. This is supported by our field experiments, wherein the number of eggs laid by the fruit flies and the number of parasitized hosts by the parasitoids decreased when weaver ants were present. This finding is consistent with the indication that indirect predation can be as costly for the forager as direct consumption in terms of fitness and population growth [13, 14, 51].

### Weaver ant effects on patch leaving tendency

Fruit flies and parasitoids differ in the factors affecting fruit leaving probability, a parameter related to patch residence duration. The encounters with ants, which can be associated with predation risk, was the main factor affecting the latency until a forager leaves the patch in both fruit fly and parasitoid species. While this was the only influential parameter for both parasitoid species, in the fruit flies *B*. *dorsalis* and *C*. *cosyra* other factors (e.g. probing activity, fruit re-visit etc.) influenced the tendency that females leave the fruit. Differences in foraging abilities and strategies between parasitoids and fruit flies may explain these divergent results. Parasitoids are slower in flying when compared to fruit flies [52]: when on the fruit, the flies were able to rapidly jump off the mango dome when encountering ants, while the parasitoids took more time, giving the ants a better chance of attacking them. Our experiments show that in trials with ants patrolling on the mango, both parasitoid species spent less time on the fruit in comparison to the fruit flies, and, when looking at multiple visits on the mango, *F*. *arisanus* was the most risk-averse species, with the smallest number of fruit re-visits. Even if from our observations a direct predation effect was small, the weaver ants were able to catch the foraging parasitoid female in three occasions for both *F*. *arisanus* and *D*. *longicaudata*, while this happened only once for the fruit fly species. Parasitoids may perceive the presence of ant workers as a high cost for foraging and adopt an evasive strategy of decreasing foraging activity in order to reduce the probability of being predated upon [4]. In general, one common reaction to increased predation risk is reduced prey activity, which implies reduced movement and/or seeking refuge [12]. Several studies show that under risk of predation, more skilful foragers can adopt riskier behaviours, such as foraging or repeatedly visiting a patch, because they are better equipped to avoid predation [10, 53, 54]. Furthermore, it must be considered that weaver ants may react differently to parasitoids and fruit flies, respectively. The different responses by the ants can in turn distinctively influence the response from the prey. Weaver ants are a honeydew-tending species, which farm and rely on Homoptera for their sugar supply [29]. In general, honeydew-tending ants have a propensity to protect their food sources, especially from natural enemies such as parasitoids [55]. Thus, parasitoids searching for fruit fly hosts may be perceived by the ants as a threat to their honeydew food sources.

After probing on the mango dome, the patch leaving tendency was reduced for both fruit fly species. In order to successfully oviposit, a female fruit fly drills into the fruit skin and penetrates the fruit pericarp with the ovipositor. This activity is quite demanding in terms of time and energy [56]. Thus, it is reasonable that a female probing on a patch may perceive it as an increased opportunity to successfully oviposit. This in turn can positively influence the female perception of patch quality and, consequently, the tendency to leave the fruit decreases.

Repeated mango visits increased patch leaving probability for *B*. *dorsalis* females, but not for *C*. *cosyra* (Table 1). According to optimal foraging theory, already visited patches can be either already exploited by the female, or already infested by other females or, when a predator is present, connected to predation risk [41, 46]. In all these cases, the forager’s perception of patch quality can be altered, with an increase in patch leaving tendency [42]. *Ceratitis cosyra* females were less likely to leave the fruit when experiencing encounters with ants in the cage. In our setup, patrolling ants were free to move both on the fruit and on the cage walls. Thus, *C*. *cosyra* females could encounter the ants both when on the mango as well as when in the cage. It is reasonable to think that when encountering the ants in the cage, in order to avoid potential predation, a female would fly back onto the fruit, thus decreasing the tendency to leave it. When females experience predation risk both in the cage and on the foraging patch (i.e. the mango), a female evaluation of the patch could change accordingly, with more time spent on the fruit where reproductive chances are higher than on the cage.

In a two-by-two species comparison between fruit fly species and parasitoid species, respectively, we did not find any difference in patch residence and oviposition time between *B*. *dorsalis* and *C*. *cosyra*, or between *F*. *arisanus* and *D*. *longicaudata*, when ants were present in the observation cage. However, in the field trials, when they foraged in the presence of ants, *C*. *cosyra* laid a higher number of eggs than *B*. *dorsalis*, while *D*. *longicaudata* had a higher parasitism rate when compared to *F*. *arisanus*. There is evidence that the two fruit fly species, as well as the two parasitoids, differ from one another in host handling and oviposition behaviour. *Bactrocera dorsalis* females take more time for probing when compared to *C*. *cosyra*. The egg-prepupal parasitoid, *F*. *arisanus* uses fruit fly eggs as hosts, and as fruit flies usually lay eggs in clutches, *F*. *arisanus* females can stay on one spot of the fruit, parasitising their host, for an average of around 134s for ovipositing [57]. Contrarily, *D*. *longicaudata* attacks the larvae as its host, and spends on average only around 30s per oviposition [58]. Rapid host-handling ability and fast oviposition might be advantageous in the presence of a predator in order to decrease predation risk, and thus increase the overall foraging success [9, 15]. The faster probing ability of *C*. *cosyra* females, and the shorter oviposition duration by *D*. *longicaudata*, might be beneficial when a predator is present, and might result in them being better competitors when compared to *B*. *dorsalis* and *F*. *arisanus*, respectively. This finding is also relevant when considering competitive interactions among the fruit fly species. From previous studies there is evidence that *B*. *dorsalis* outcompetes *C*. *cosyra* in the field [24, 59]. As *C*. *cosyra* is more successful in infesting mangoes in the presence of weaver ants, predator presence may favour the native fruit fly over the invasive one, reverting the effect of competitive dynamics between the two species. There is evidence that predation can alter competitive interactions among the species within an ecological community [4, 12].

### Conclusions

In conclusion, what could be the consequences of weaver ants’ indirect effect on parasitoid-host dynamics at population level? Predation risk can affect population growth of the prey even if the prey is not killed and consumed by the predator [44, 60]. Even though direct killing of adult fruit flies and parasitoids by weaver ants is rare, our findings demonstrate that as generalist predators, weaver ants hamper both fruit fly and parasitoid foraging behaviour through indirect predation. As for both parasitoids and fruit flies, oviposition success is intrinsically linked to foraging behaviour, the negative effects due to weaver ants may reduce the fitness of these prey organisms. In the presence of the weaver ants, foraging efficiency was more affected in the parasitoids than in the fruit flies. Intraguild predation models assume that the presence of an intraguild predator has a negative effect on the intraguild prey. Intraguild predation theory predicts that the intraguild prey will be excluded when in presence of the intraguild predator, if the latter is a superior competitor for the shared prey [4]. As in this system, the presence of an intraguild predator such as the weaver ants reduces the time that the parasitoids spent searching for hosts on the fruit, which may lead to a reduction in parasitoid population growth, potentially causing an exclusion of the parasitoids in areas where weaver ants are present. Thus, parasitism efficiency on the fruit fly hosts may be impaired. The negative effect that generalist predators may have on parasitoids have been reported in several field studies. Observation studies have, for example, shown that Argentine ants, a generalist ground-dwelling predator species, were reducing the population density of two parasitoid species affecting San Jose scale in the field [61]. However, the negative effect of the weaver ants on the parasitoids might not necessarily result in an increase in the fruit fly population, as weaver ants alone can reduce fruit fly population levels. In fact, our results show that, in the presence of weaver ants, *B*. *dorsalis* suffered the highest reduction of eggs laid in the fruit. Thus, it is reasonable to think that the weaver ants may reduce the fruit fly population growth.

Our study highlights how a behavioural investigation within a multi-trophic system may be important to understand the underlying forces shaping the trophic interactions within a community, contributing to formulate predictions on population dynamics in such systems. As our system involves species of economic importance for biological control, our findings may also be considered when planning for the implementation of biological control programs involving multiple control agents.

## Supporting Information

S1 Dataset Field cage experiment data. (XLSX)

S2 Dataset Observation cage experiment data. (XLSX)

S3 Dataset Observation cage experiment data for Cox analysis. (XLSX)
